# Neuropsychological and Psychiatric Outcomes in Dextro-Transposition of the Great Arteries across the Lifespan: A State-of-the-Art Review

**DOI:** 10.3389/fped.2017.00059

**Published:** 2017-03-24

**Authors:** Leila Kasmi, Damien Bonnet, Michèle Montreuil, David Kalfa, Nikoletta Geronikola, David C. Bellinger, Johanna Calderon

**Affiliations:** ^1^Laboratory of Psychopathology and Neuropsychology, Department of Psychology, University Paris 8, Paris Lumières – CNRS, Saint-Denis, France; ^2^Referral Center for Complex Congenital Cardiac Malformations, Department of Congenital and Pediatric Cardiology, Necker Hospital, University Paris Descartes, Sorbonne Paris Cité, Paris, France; ^3^Division of Cardiac, Thoracic, and Vascular Surgery, Section of Pediatric Cardiac Surgery, Morgan Stanley Children’s Hospital of New York-Presbyterian, Columbia University Irving Medical Center, Columbia University, New York, NY, USA; ^4^Department of Neurology, Boston Children’s Hospital, Harvard Medical School, Boston, MA, USA; ^5^Department of Psychiatry, Boston Children’s Hospital, Harvard Medical School, Boston, MA, USA

**Keywords:** dextro-transposition of the great arteries, neuropsychological outcomes, psychiatric disorders, cognitive, executive function, open-heart surgery

## Abstract

Advances in prenatal diagnosis, perioperative management, and postoperative care have dramatically increased the population of survivors of neonatal and infant heart surgery. The high survival rate of these patients into adulthood has exposed the alarming prevalence of long-term neuropsychological and psychiatric morbidities. Dextro-transposition of the great arteries (d-TGA) is one of the most extensively studied cyanotic congenital heart defect (CHD) with regard to neurodevelopmental outcomes. Landmark studies have described a common neurodevelopmental and behavioral phenotype associated with d-TGA. Children with d-TGA display impairments in key neurocognitive areas, including visual–spatial and fine motor abilities, executive functioning, processing speed, and social cognition. As they grow older, they may face additional challenges with a worsening of deficits in higher order cognitive skills, problems in psychosocial adjustment and a higher-than-expected rate of psychiatric disorders, such as attention-deficit hyperactivity disorder, depression, and anxiety. The aim of this review is to summarize the available recent data on neuropsychological and psychiatric outcomes in individuals with d-TGA after the arterial switch operation. We present findings within a life-span perspective, with a particular emphasis on the emerging literature on adolescent and young adult outcomes. Finally, we propose avenues for future research in the CHD adult neuropsychology field. Among these avenues, we explore the potential mechanisms by which pediatric neurodevelopmental impairments may have lifelong adverse effects as well as alternative interventions that could optimize outcomes.

## Introduction

Dextro-transposition of the great arteries (d-TGA) accounts for 5–7% of congenital heart defects (CHD), with a prevalence of 0.2 per 1,000 live births (1, 2). Individuals with d-TGA represent a unique and relatively homogeneous study cohort with the Arterial Switch Operation (ASO) being now the standard-of-care. Moreover, d-TGA is infrequently associated with extra-cardiac anomalies, including genetic abnormalities, reducing potential confounding variables in follow-up studies. Since the first successful ASO in 1975 (3), survival rates have significantly increased, resulting in a demographic shift: adults now outnumber children living with d-TGA (4, 5). Long-term outcomes in d-TGA, and in CHD population as a whole, pose a public health challenge for screening, diagnosis, and treatment. The aim of this state-of-the-art review is to integrate recent data on neuropsychological and psychiatric outcomes in d-TGA after the ASO within a life-span perspective. Finally, we propose avenues for future research, including discussion on the potential mechanisms of long-term impairments and interventions to optimize outcomes.

## Neuropsychological Outcomes in Children with d-TGA

Much of our knowledge on the neuropsychological profile of children with CHD comes from decades of study of survivors of d-TGA. Although the prevalence of severe neurological disorders is very low in this population (6), neurodevelopmental impairments are consistently reported (7). Early development is characterized by mild to moderate delays in cognitive, motor, and language function, with scores on the Bayley Scales of Infant Development (BSID) 0.5–1 SD below the expected mean values (8–11). The Boston Circulatory Arrest Study (BCAS) offers one of the most comprehensive analyses of the neuropsychological phenotype of these patients (9, 12–14). This longitudinal prospective study followed-up children from infancy to adolescence. At 12 months, 20% of infants achieved a psychomotor score ≤80 on the BSID and were less vocal than expected, suggesting delays in expressive language development (9). Recent findings reported an improvement on early outcomes for infants with d-TGA. Andropoulos et al. (15) reported mean Cognitive scores on the BSID within the normal population range, although Language and Motor scores were 7–10 points lower than the expected means. Although evaluations in infancy are important for early interventions, they are not strongly predictive of long-term scores (16), which may lead to false negatives.

Several studies have characterized the cognitive outcomes of children with d-TGA. It has become clear that intelligence abilities, as measured by Full-scale IQ scores, are generally within the normal range (6, 17). Nevertheless, deficits are often apparent in specific neurocognitive areas. Speech and motor impairments were reported in both European (17, 18) and North-American studies (13, 19). Bellinger et al. (12, 13) reported below age-expected scores for 4- and 8-year-old children in visual–spatial skills, academic achievement, working memory, hypothesis generation, sustained attention, and higher order language skills. In general, lower-level skills were relatively intact, but children had difficulty integrating or coordinating these skills to achieve higher order goals (13).

There is growing awareness that executive functioning is particularly vulnerable in d-TGA (20–22). In the BCAS, 8-year children had substantial difficulties in metacognitive aspects of behavior, such as planning, organizational skills, and cognitive flexibility (13). Impairments were evident on both verbal and non-verbal tasks, with children tending to focus on isolated details at the expense of a coherent organization of elements (13, 23). Calderon et al. (24, 25) corroborated these findings in two cohorts of 5- and 7-year olds with d-TGA. In these studies, children had difficulties elaborating a strategy to achieve a goal, i.e., anticipating the right number of actions to reproduce a visual model. They also had deficits in attentional control and inhibition of automatic tendencies, as well as lower working memory. Executive functioning issues start early in preschool years. Calderon et al. (25) demonstrated that executive function impairments were common at the age of 5, in tasks measuring behavioral control, attention, working memory, and cognitive flexibility. Executive function deficits were also reported in children with other types of complex CHD (26, 27), suggesting that they are part of the “developmental signature” of critical CHD.

Recently, deficits in social cognition were described in children with d-TGA (24, 28, 29) manifested by difficulties to interpret social stimuli and situations, e.g., facial emotional expressions, self- and other’s intentions. A significant proportion had difficulties in identifying the emotional and cognitive states of others (Theory of Mind deficits).

In sum, the cognitive and behavioral challenges faced by children with d-TGA place them at high risk of long-term learning disabilities and academic under-achievement (22). Indeed, nearly 50% requires early remedial services (e.g., psychotherapy, speech therapy, or educational support) (30).

## Adolescents with d-TGA: Cognitive and Psychiatric Outcomes

### Cognitive Outcomes

To our knowledge, only two studies focused on the cognitive outcomes of adolescents with d-TGA corrected by ASO (14, 31). In the BCAS, 139 adolescents with d-TGA (16.1 ± 0.5 years old) were evaluated (14). Patients’ mean scores were lower than the expected population means on tests assessing academic skills, visuo-spatial skills, long-term memory, executive functions, and social cognition. Frequencies of scores ≥1 SD or ≥2 SD below the normative mean were, respectively, 26 and 7% for academic skills composite, 35 and 17% for memory index, and 54 and 20% for visuo-spatial index (compared to the expected frequencies of 16 and 2%, respectively). By parent report, about one in five patients had attention or executive impairments in daily life. In the Aachen Study (17, 31, 32), 60 patients who underwent the ASO were followed-up from preschool to adolescence (16.9 ± 1.7 years old). Adolescents’ IQ scores were not reduced compared to the population norms, but the frequency of IQ scores ≥2 SD below the expected mean was increased, especially for Performance IQ (11%) (31).

Some study cohorts have included adolescents with d-TGA as well as other types of CHD (33–37), but investigations directly comparing the d-TGA and other CHD groups are scarce. In the study of Matos et al. (34), adolescents with cyanotic CHD scored lower than adolescents with acyanotic CHD on all cognitive domains assessed, although the difference was significant only for visuo-constructive abilities. Cassidy et al. (33) evaluated executive function in 352 adolescents with cyanotic CHD (d-TGA, TOF or single-ventricle anatomy requiring Fontan procedure) and 111 controls. d-TGA, TOF, and Fontan groups had lower performances than controls on cognitive flexibility and verbally mediated executive function skills. Only visuo-spatially mediated skills were higher in the d-TGA group compared to the other groups with CHD. Despite the relative preservation of these abilities in the d-TGA group, impairment (score ≥ 1.5 SD below the expected mean) was twice as frequent when compared to controls.

In sum, we observe a continuum between cognitive impairments observed in children and those detected in adolescents with d-TGA. These issues, which may increase with age (13, 14, 25), involve diverse domains but mostly attention, visuo-spatial abilities, and executive functions.

### Psychosocial and Psychiatric Outcomes

Adolescence is a vulnerable time, when a variety of psychosocial and psychiatric problems emerge (38). Successful transition from childhood to adolescence depends on the development of effective self-management skills and a sense of autonomy (7). For individuals with CHD, it is also an opportunity for intervention before transitioning to adult health care (39). This is important, as mental health problems can predict future adjustment difficulties, such as unemployment, risk-taking behaviors, substance abuse, and suicidality (40). Although many adolescents with d-TGA do not have residual cardiac morbidities or health chronic conditions, they may still be at risk of poor psychosocial outcomes and mental health problems. Several studies identified increased incidence of internalizing problems (i.e., anxiety, somatic complaints, depressive symptoms) in adolescents with complex CHD when compared to general population (40–43). Externalizing problems were also consistently reported, on both parent and self-report measures (41, 44). However, because these studies typically include mixed types of CHD, it is difficult to draw inferences about the risk among individuals with d-TGA.

Few studies reported specifically on adolescents with d-TGA. Hövels-Gürich et al. (42) and Karl et al. (19) showed an increased risk of parent-reported psychosocial maladjustment in children and adolescents with d-TGA. In the BCAS, 16-year-olds with d-TGA were more likely than controls (35 versus 20%) to meet criteria for a lifetime psychiatric diagnosis, as evaluated by the Schedule for Affective Disorders and Schizophrenia for School-aged Children (K-SADS) (45). They also had a greater proportion with Attention Deficit Hyperactivity Disorder (ADHD), both lifetime (19 versus 7% for referents) and current (16 versus 3% for referents). However, the frequencies of mood or anxiety disorders did not differ between groups. On the Children’s Global Assessment Scale, which assesses psychosocial functioning in different life-settings, adolescents with d-TGA obtained poorer scores, and 15% fell within the range of pathological functioning. Parent- and self-reports of psychiatric symptoms in the d-TGA group also identified more depressive, anxiety, and post-traumatic stress symptoms. Impaired cognitive functioning and greater parental stress at 8 years were the strongest predictors of poor psychosocial and psychiatric outcomes at 16 years. Of note, the prevalence of psychiatric disorders in d-TGA is lower compared to other forms of critical CHD such as single-ventricle physiology (46). However, the rates of mental health disorders in d-TGA are much higher than those reported in the national US population (47).

Finally, neuropsychological and mental health morbidities can impede successful transition from pediatric to adult health care (7). Studies showed that adolescents with CHD struggle to successfully transit to adult care and assume control of their health-care management (48–50). Focused psychosocial care, including strategies for managing health and coping with medical decision-making, should be a key facet of the transition process (51). According to the American Heart Association’s transition guidelines (39), a standard core educational curriculum should include topics related to lifestyle issues including learning disabilities, anxiety, depression, and high-risk behaviors. The transition process must also include facilitated access to mental health services (50).

## Adults with d-TGA: Emerging Evidence

Despite the increased number of adults with d-TGA after the ASO (52), studies on their cognitive or psychological outcomes are infrequent. Thus, to our knowledge, no studies have focused specifically on the cognitive outcomes of adults who underwent the ASO. Two recent studies (53, 54) investigated the neuropsychological outcomes of adults with CHD, including d-TGA. However, in these cohorts, most of the adults with d-TGA had not undergone the ASO but the atrial switch procedure (operation often conducted in this population before the development of the ASO). In the study of Klouda et al. (53), adults with critical CHD (i.e., d-TGA or Fontan, *n* = 24, 32.8 ± 7.6 years old) had lower scores than expected in multiple domains: psychomotor speed, processing speed, sustained and executive attention, and on the overall, neurocognitive index. Tyagi et al. (54) observed that d-TGA adults (*n* = 80, 19–50 years old) scored worse than those with mild CHD (*n* = 84) on an overall neuropsychological index. Furthermore, proportions with cognitive impairments on at least three tests were higher in the d-TGA group (49%) compared to the mild CHD group (26%). Most of the impairments observed in CHD groups involved divided attention, executive functions, and fine motor function.

To our knowledge, no studies have investigated psychiatric risks specifically in adults who underwent the ASO. Two studies evaluated the psychological outcomes of adults who underwent the atrial switch procedure (55, 56), finding that approximately 20% met criteria for a psychiatric disorder. Studies comparing results between cardiac diagnoses showed that psychological outcomes were worse among adults with cyanotic or complex CHD than among adults with acyanotic or mild CHD (41, 57, 58). Findings from mixed cohorts of adults with CHD show high rates of psychiatric disorders (59–62), with about 30% meeting diagnostic criteria for at least one lifetime mood disorder (i.e., major depressive disorder, bipolar disorder, etc.), and 26–28% for at least one anxiety disorder (i.e., generalized anxiety, social phobia, etc.) (61, 62). Findings on the frequency and severity of current anxiety-depressive symptoms assessed by self-report are mixed. Some studies found high symptomatology in adults with CHD (59, 61, 63–65), whereas others did not (66–72). According to several authors (60, 71–74), denial mechanisms or coping strategies are frequently used by CHD patients and could contribute to the favorable psychological outcomes sometimes suggested by self-assessments.

Many CHD patients with psychiatric disorders appear not to receive adequate treatment. Kovacs et al. (61) found that 69% of patients with a mood or anxiety disorder did not receive psychotherapy or psychotropic drugs. Other studies report that only 11–12% of patients receive psychological counseling, despite high rates of anxiety-depressive syndromes (62, 64). In adults with CHD, presence of a high depressive or anxiety symptomatology is associated with higher rates of unemployment (64), lower quality of life (QoL) (62, 64, 68), greater consumption of tobacco and alcohol (75), poorer adherence to treatment (76), and worse cardiac prognosis (65).

In summary, to date, few data are available on the cognitive and psychiatric outcomes of d-TGA adults after the ASO. Elevated risk of attentional and executive impairments and of mood and anxiety disorders may be prevalent. More research is needed to better understand the long-term cognitive and psychological trajectory of these adults and to investigate how cognitive and psychiatric disorders influence social interactions, employability, and achievement.

## Futures Avenues

### Potential Mechanisms for Long-term Impairment

The mechanisms for lifelong cognitive and psychosocial difficulties in CHD are multifactorial. Early cognitive deficits play a decisive role (7). In children with d-TGA, lower full-scale IQ is associated with lower parent-reported psychosocial QoL (77). Moreover, as reported in other pediatric populations, such as children born preterm (78), a cascade of effects may be observed, where early deficits mediate the expression of new symptoms and/or the worsening of pre-existing impairments. In children with d-TGA, Calderon et al. (25) showed that deficits on some aspects of executive functioning (i.e., cognitive flexibility) became worse between the ages of 5 and 7, whereas more basic processes (e.g., motor control) tended to catch-up. Interestingly, Cassidy et al. (22) reported that reading and math difficulties of adolescents with d-TGA at age 16 were predicted by deficits in processing speed and executive function at age 8. Indeed, executive function deficits and difficulties in other key areas of neurodevelopment are often comorbid. In d-TGA, poor working memory adversely impacted children’s language comprehension and mathematic skills (13) and poor inhibitory control was associated with deficits in social cognition (24). These weaknesses might result in more severe dysfunction as expectations increase with age. Patients with these cognitive challenges may find establishing and maintaining social relationships difficult, especially in adolescence and adulthood (20). Peer interactions and social cues (e.g., body language, irony, sarcasm) become more complex, making decoding them difficult to individuals with d-TGA. These and other cognitive deficits may “derail” the developmental trajectory in the mental health domain. Of note, executive function deficits increase the risk of psychosocial and psychiatric morbidities in adolescents with d-TGA. In BCAS, worse psychosocial outcomes and poorer QoL of 16-year olds were strongly predicted by executive dysfunction, suggesting a robust association between these higher order processes and self-perception and psychosocial adaptation (45).

Finally, it is possible that accumulating cognitive deficits, particularly in organization, flexibility, and self-control, along with poor social interactions trigger a lower threshold for stress, predisposing individuals to anxiety and depression. This hypothesis is, however, speculative and more research is needed.

### Interventions to Improve Outcomes

**Figure 1:**
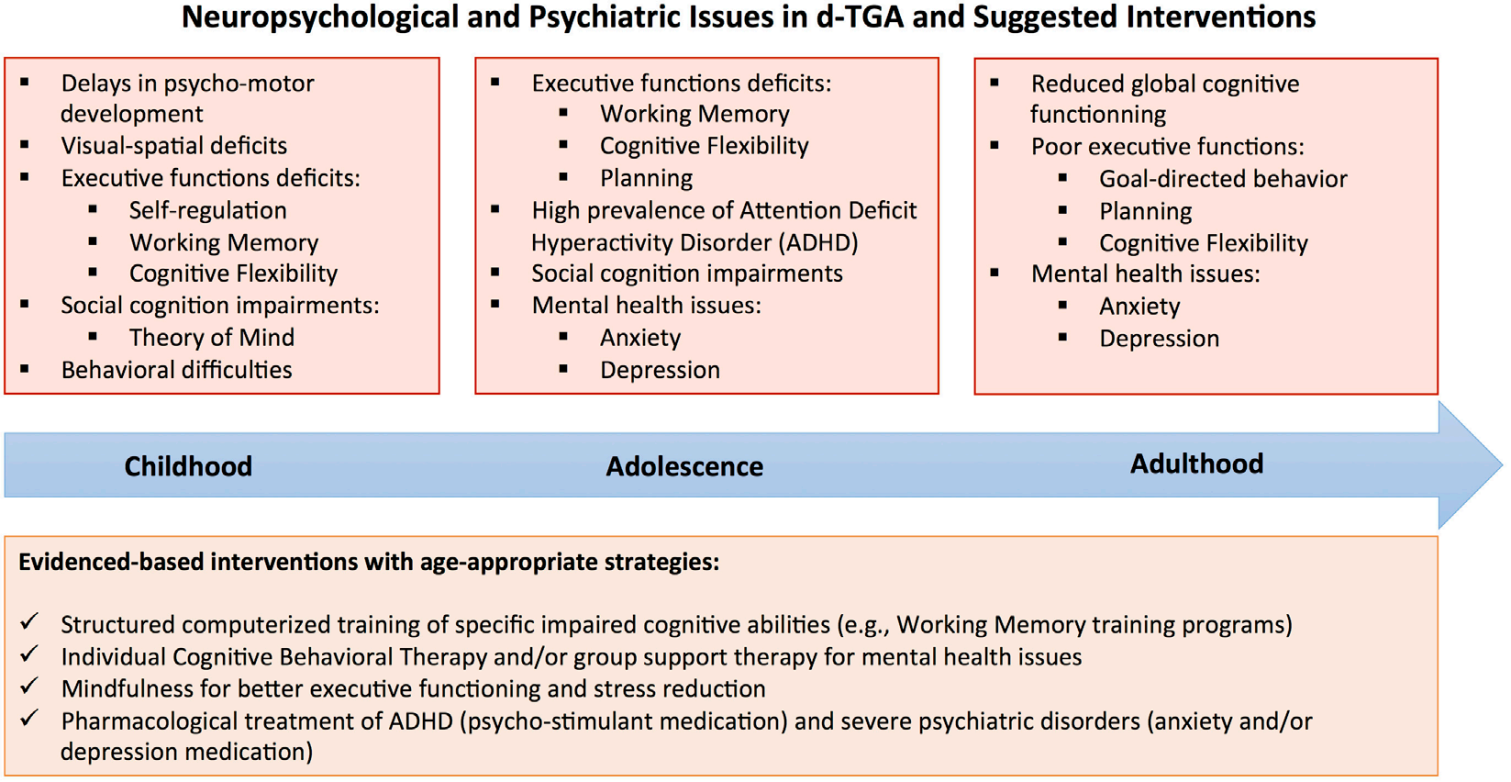
**Neuropsychological and psychiatric issues in dextro-transposition of the great arteries (d-TGA) by age group and suggested interventions**.

As a result of progress in understanding the challenges patients with CHD face, interventions to prevent, mitigate, or palliate morbidities are emerging (Figure 1). Many hospital-based Cardiac Neurodevelopmental Programs are providing early screening and treatment recommendations. Nevertheless, there is urgent need for well-designed randomized controlled trials evaluating the efficacy of neurocognitive and psychological interventions in this population. McCusker et al. (79) conducted a study evaluating the efficacy of interventions focused on maternal and family functioning, individualized psycho-education, and outreach to community health-care providers. Although maternal mental health and family functioning improved, few benefits were seen on children’s behavioral outcomes and school achievement.

One major concern in the d-TGA population relates to executive dysfunction, and thus, a proof-of-concept for evidence-based interventions is needed (20). Many behavioral interventions may be implemented from early childhood and throughout the lifespan. Intensive computerized training targeting attention and working memory has improved executive skills and behavioral outcomes in children with ADHD (80) and children born with low birth weight (81). If these programs prove effective, reduction of executive function morbidities could reduce the likelihood that psychiatric disorders emerge (82, 83). Research is ongoing to determine the short- and long-term effects of such structured interventions in youth with CHD.

As recommended by several Associations (39, 84), many transitional and educational programs for patients with CHD are being developed. It may be useful to integrate psychologists into these medico-social programs. Their intervention, within the framework of individual or group support, could prevent subclinical disorders from evolving into depression or anxiety disorders. These interventions could focus on the emotional expression and management, and the development of effective coping strategies. Interventions aiming to optimize the “sense of coherence” (85), that is, the understanding of events, the confidence in one’s power and resources, and the ability to give sense to events, could also improve the psychosocial outcomes of patients with CHD.

Finally, several mental health treatments, including pharmacotherapy and psychotherapy, are available and could be tested in this population. Cognitive behavioral therapy, targeting the modification of maladaptive thought patterns and behaviors, has proven to be effective in treating anxiety and depression disorders (86). Mindfulness and relaxation techniques may also be beneficial. Mindfulness training is associated with substantial reduction in stress-related (82), depressive (83) and ADHD symptoms (87).

## Conclusion

Over the last two decades, dramatic progress was made in the understanding of neuropsychological and psychiatric outcomes of individuals with d-TGA (for a summary of main studies, please refer to Table 1). The cardiac neurodevelopmental and mental health field is moving from a descriptive approach of challenges to an in-depth understanding of neurobiological and psychological mechanisms of impairment. Novel hypotheses will be critical to improve outcomes and QoL for individuals with d-TGA.

**Table 1:** **Overview of selected studies on neuropsychological and psychiatric outcomes for patients with d-TGA**.

Reference	*n*, age (years), diagnosis	Neurocognitive or psychiatric assessment	Main results
**Children**			
Bellinger et al. (12)	*n* = 158, 4 years, d-TGA	– WPPSI revised, – Peabody Developmental Motor Scales, – Grooved pegboard, – Test for auditory comprehension of language, – Receptive one-word picture vocabulary test, – Expressive one-word picture vocabulary test, – Illinois test of psycholinguistic abilities.	Lower than expected mean scores in general intelligence (IQ), expressive language, visual-motor integration, motor function, and oromotor control.

Bellinger et al. (13)	*n* = 155, 8 years, d-TGA	– WISC III, – WIAT, – Wide range assessment of memory and learning, – Developmental test of visual–motor integration, – Test of variables of attention, – Rey–Osterrieth complex figure, – Verbal fluency, – Wisconsin card sorting test, – Trail making test, – Formulated sentences subtest of the clinical evaluation of language fundamentals, – Controlled oral word association test, – Grooved pegboard.	Lower than expected scores in academic achievement, memory, visual-spatial skills, sustained attention, and higher order language skills. Higher than expected proportion with scores >1SD below normative values in executive functions (e.g., planning, cognitive flexibility).

Calderon et al. (24)	*n* = 21, 7 years, d-TGA	– Columbia Mental Maturity Scale, – Animal Stroop test, – Statue subtest from the NEPSY, – Tower of London, – Digit span, – Corsi block-tapping task, – False belief tasks (1st and 2nd order).	Patients’ mean IQ scores within the normal range. Compared to a control group, patients with d-TGA had lower scores in executive functions (i.e., inhibition, working memory, planning) and social cognition (i.e., theory of mind).

Calderon et al. (25)	*n* = 45, 5/7 years, d-TGA	– Columbia Mental Maturity Scale, – Comprehension subtest from the NEPSY, – Digit span, – Spatial span, – The hand game, – Hearts and flowers incongruent and mixed conditions, – Day and night, – Animal Stroop test, – Dimensional change card sorting test.	Patients’ mean scores lower than controls’ mean scores in receptive language, attention, and executive functions (i.e., inhibition, cognitive flexibility). Persistent impairments in cognitive inhibition and cognitive flexibility from ages 5 to 7.

Freed et al. (8)	*n* = 82, 1.5–2 years, d-TGA	BSID II	Most patients with scores > 1SD below normative values in cognitive, motor, and language function.

Hicks et al. (11)	*n* = 91, 2 years, d-TGA	BSID III	Higher than expected proportion of patients with scores >1SD or >2SD below normative values in language function.

Hövels-Gürich et al. (32)	*n* = 77, 3–9 years, d-TGA	– K-ABC, – Vocabulary subtest of the K-ABC, – Kiphard and Schilling body coordination test, – Denver developmental screening test, – Frostig developmental test of visual perception.	Patients’ mean IQ scores within the normal range. Lower than expected scores in motor function, vocabulary, and acquired abilities.

Hövels-Gürich et al. (17)	*n* = 60, 7–14 years, d-TGA	– K-ABC, – Kiphard and Schilling body coordination test, – Oral and speech motor control test, – Mayo tests of speech and oral apraxia, – Illinois test of psycholinguistic abilities, – Test of auditory analysis skills.	Speech, motor, and developmental impairments more frequent compared to the general population. Lower than expected scores in acquired abilities and language.

Hövels-Gürich et al. (42)	*n* = 60, 7–14 years, d-TGA	Achenbach child behavior checklist	Parent-reported psychosocial maladjustment more frequent than in the general population on all domains (i.e., internalizing, externalizing, social, and attention problems, and competences).

Karl et al. (19)	*n* = 74, 4–14 years, d-TGA	– WPPSI, – WISC III, – Movement Assessment Battery, – Achenbach child development checklist, – Achenbach teacher report form.	Patients’ mean IQ scores within the normal range. Lower scores in motor function. Parent- and teacher-reported psychosocial maladjustment more frequent than in a control group on domains including behavioral, speech, language, and learning ability problems.

McGrath et al. (16)	*n* = 135, 1/8 years, d-TGA	*Evaluation at 1 year* – BSID, – Fagan test of infant intelligence. *Evaluation at 8 years* – WISC III, – WIAT.	Most 1-year test scores were statistically but modestly associated with 8-year test scores. The majority of patients with scores >1SD below normative values at 8 years had displayed scores >1SD at 1 year.

**Adolescents**			

Bellinger et al. (14)	*n* = 139, 16 years, d-TGA	– WIAT II, – General Memory Index of the Children’s Memory Scale, – Test of visual–perceptual skills, – Rey–Osterrieth complex figure, – Delis–Kaplan executive function system, – Behavior rating inventory of executive function (child, parent, and teacher versions), – Connors attention-deficit and hyperactivity disorder (parent version), – Reading the mind in the eyes test, revised.	Lower than expected scores on academic skills, visuo-spatial skills, memory, executive functions, and social cognition. Higher than expected proportion of patients with scores >1SD or >2SD below normative values in academic skills, memory and visuo-spatial skills. By parent reports, about 1 in 5 had attention or executive impairments in daily life.

Cassidy et al. (22)	*n* = 139, 8/16 years, d-TGA	– WISC III, – WIAT II, – Trail making test, – Test of variables of attention.	Processing speed associated with executive functions (i.e., working memory, inhibition, and shifting) and academic skills at 8 and 16 years.

DeMaso et al. (45)	*n* = 139, 16 years, d-TGA	– Schedule for affective disorders and schizophrenia for school-aged children, – Children’s Global Assessment Scale, – Brief Psychiatric Rating Scale for Children, – Children’s Depression Inventory, – Revised Children’s Manifest Anxiety Scale, – Child stress disorders checklist, – Posttraumatic Stress Diagnostic Scale, – Conners’ attention-deficit/hyperactivity disorder rating scales, – Conduct Disorder Scale.	Patients were more likely than controls to meet criteria for a lifetime psychiatric diagnosis. Higher lifetime and current prevalence of attention-deficit/hyperactivity disorder. Psychosocial functioning was within a pathological range for 15% of patients. Parent- and self-reports identified high depressive, anxiety, and posttraumatic stress symptoms.

Heinrichs et al. (31)	*n* = 60, 14–21 years, d-TGA	– Hamburg-Wechsler intelligence test, – Analytical thinking subtests of the Leistungsprüfsytem nach Horn, – Mannheimer Rechtschreib test.	Patients’ mean IQ scores within the normal range. Higher than expected proportion of patients with IQ scores ≥2. Lower than expected scores on orthography.

**Adults**			

Klouda et al. (53)	Total mixed cohort, *n* = 48, 18–49 years *n* = 24, moderate CHD, *n* = 24, complex CHD (including d-TGA, *n* = 9).	CNS vital signs	Patients with critical CHD had lower than expected scores on multiple domains: psychomotor speed, processing speed, sustained and executive attention, and on the overall, neurocognitive index.

Tyagi et al. (54)	Total mixed cohort, *n* = 310, 18–76 years *n* = 80, d-TGA, *n* = 81, TOF, *n* = 65, SV anatomy, *n* = 84, mild CHD.	– Controlled oral word association test, – Grooved pegboard, – Rey auditory verbal learning test, – Stroop test, – Symbol digit modalities test, – Trail making test, – Wisconsin card sorting test, – Wechsler Adult Intelligence Scale.	d-TGA patients scored worse than those with mild CHD on an overall neuropsychological index. Proportion with scores >1.5 SD below normative values on at least 3 tests was higher in the d-TGA group compared to both the mild CHD group and the expected frequency in the general population.

van Rijen et al. (56)	Total mixed cohort, *n* = 349, 20–46 years, *n* = 55, d-TGA, *n* = 72, TOF, *n* = 37 pulmonary stenosis, *n* = 185, mild CHD.	– Young adult self-report, – Young adult behavior checklist.	Diagnosis of d-TGA was associated with higher risks of behavioral problems, particularly externalizing problems (e.g., intrusive and aggressive behavior).

*BSID, Bayley Scales of Infant Development; CHD, congenital heart defects; d-TGA, dextro-transposition of the great arteries; IQ, intelligence quotient; K-ABC, Kaufman assessment battery for children; SV, single ventricle; TOF, tetralogy of Fallot; WIAT, Wechsler individual achievement test; WISC, Wechsler intelligence scale for children; WPPSI, Wechsler preschool and primary scale of intelligence*.

## Author Contributions

LK and JC conducted the literature search, drafted this manuscript, and revised it critically for important intellectual content. DB, MM, DK, NG and DCB made substantial contributions to the conception of this review and revised it critically for important intellectual content. All the authors (LK, DB, MM, DK, NG, DCB, and JC) approved the final version of the manuscript and agreed to be accountable for all aspects of the work in ensuring that questions related to the accuracy or integrity of any part of the work are appropriately investigated and resolved.

## Conflict of Interest Statement

The authors declare that the research was conducted in the absence of any commercial or financial relationships that could be construed as a potential conflict of interest. The reviewer MN declared a shared affiliation, though no other collaboration, with the authors DB & JC to the handling Editor, who ensured that the process nevertheless met the standards of a fair and objective review.
